# Convergence between Regulation of Carbon Utilization and Catabolic Repression in *Xanthophyllomyces dendrorhous*

**DOI:** 10.1128/mSphere.00065-20

**Published:** 2020-04-01

**Authors:** Pilar Martinez-Moya, Sebastián Campusano, Pamela Córdova, Alberto Paradela, Dionisia Sepulveda, Jennifer Alcaíno, Marcelo Baeza, Víctor Cifuentes

**Affiliations:** a Departamento de Ciencias Ecológicas, Facultad de Ciencias, Universidad de Chile, Santiago, Chile; b Laboratorio de Proteómica, Centro Nacional de Biotecnología, C.S.I.C., Madrid, Spain; University of Georgia

**Keywords:** iTRAQ, RNA-seq, Mig1, Cyc8, Tup1, glucose, maltose, succinate, catabolic repression, *X. dendrorhous*

## Abstract

Xanthophyllomyces dendrorhous is a carotenogenic yeast with a singular metabolic capacity to produce astaxanthin, a valuable antioxidant pigment. This yeast can assimilate several carbon sources and sustain fermentation even under aerobic conditions. Since astaxanthin biosynthesis is affected by the carbon source, the study of carotenogenesis regulatory mechanisms is key for improving astaxanthin yield in X. dendrorhous. This study aimed to elucidate the regulation of the metabolism of different carbon sources and the phenomenon of catabolic repression in this yeast. To this end, protein and transcript levels were quantified by iTRAQ (isobaric tags for relative and absolute quantification) and transcriptomic sequencing (RNA-seq) in the wild-type strain under conditions of glucose, maltose, or succinate treatment and in the mutant strains for genes *MIG1*, *CYC8*, and *TUP1* under conditions of glucose treatment. Alternative carbon sources such as maltose and succinate affected the relative abundances of 14% of the wild-type proteins, which were mainly grouped into the carbohydrate metabolism category, with the glycolysis/gluconeogenesis and citrate cycle pathways being the most highly represented pathways. Each mutant strain showed significant proteomic profile changes, affecting approximately 2% of the total proteins identified, compared to the wild-type strain under glucose treatment conditions. Similarly to the results seen with the alternative carbon sources, the changes in the mutant strains mainly affected carbohydrate metabolism, with glycolysis/gluconeogenesis and the pentose phosphate and citrate cycle pathways being the most highly represented pathways. Our results showed convergence between carbon assimilation and catabolic repression in the strains studied. Interestingly, indications of cooperative, opposing, and overlapping processes during catabolic regulation were found. We also identified target proteins of the regulatory processes, reinforcing the likelihood of catabolic repression at the posttranscriptional level.

**IMPORTANCE** The conditions affecting catabolic regulation in X. dendrorhous are complex and suggest the presence of an alternative mechanism of regulation. The repressors Mig1, Cyc8, and Tup1 are essential elements for the regulation of the use of glucose and other carbon sources. All play different roles but, depending on the growth conditions, can work in convergent, synergistic, and complementary ways to use carbon sources and to regulate other targets for yeast metabolism. Our results reinforced the belief that further studies in X. dendrorhous are needed to clarify a specific regulatory mechanism at the domain level of the repressors as well as its relationship with those of other metabolic repressors, i.e., the stress response, to elucidate carotenogenic regulation at the transcriptomic and proteomic levels in this yeast.

## Abstract (precis)

The conditions affecting catabolic regulation in X. dendrorhous are complex and suggest the presence of an alternative mechanism of regulation. The repressors Mig1, Cyc8, and Tup1 are essential elements for the regulation of the use of glucose and other carbon sources. All play different roles but, depending on the growth conditions, can work in convergent, synergistic, and complementary ways to use carbon sources and to regulate other targets for yeast metabolism. Our results reinforced the belief that further studies in X. dendrorhous are needed to clarify a specific regulatory mechanism at the domain level of the repressors as well as its relationship with those of other metabolic repressors, i.e., the stress response, to elucidate carotenogenic regulation at the transcriptomic and proteomic levels in this yeast.

## INTRODUCTION

The yeast Xanthophyllomyces dendrorhous has been studied mainly for its capacity to produce astaxanthin, a pigment of commercial interest (1). Astaxanthin has been used as a feed and food pigment in the aquaculture industry and has been evaluated as a pharmaceutical component due to its antioxidant properties (1, 2). Because the natural habitat of X. dendrorhous is usually represented by tree exudates rich in antifungal substances and reactive oxygen species (ROS), it is believed that the main functions of the pigments produced by this yeast include antioxidant and protective roles against these agents (3, 4). This hypothesis is supported by the fact that this yeast has low levels of catalase activity and possesses only Mn-superoxide dismutase, lacking other antioxidant enzymes present in most yeasts (5).

X. dendrorhous can grow in the presence of various carbon sources, including glucose, sucrose, maltose, xylose, starch, succinate, glycerol, and ethanol (3–7). Notably, X. dendrorhous is the only carotenogenic yeast capable of efficiently fermenting sugars (1, 2, 5). This yeast can undergo two types of metabolism depending on the carbon source. Sugars such as glucose and fructose are oxidized by the glycolytic pathway to yield pyruvate, which is subsequently converted to ethanol even in the presence of oxygen. However, nonfermentable carbon sources, such as succinate, are metabolized via the Krebs cycle (5, 6).

The carotenoid biosynthesis pathway is largely known (2, 8, 9). Previous studies have demonstrated that the carbon source has an effect on carotenoid production in X. dendrorhous (5, 6, 8). Regarding the above, it has been shown that the level of production of carotenoids is lower when the yeast is cultivated in the presence of fermentable carbon sources and increases when it is cultivated with nonfermentable carbon sources (5, 6, 8). In addition, it has been observed that when yeast is cultivated with glucose as the sole carbon source, carotenoid synthesis starts only when the sugar in the culture medium has been depleted (5, 6).

Studies of the regulatory mechanisms of carotenogenesis have shown that the catabolic regulator Mig1 is involved in the regulation of the carotenogenic process mainly at the transcriptional level, causing the repression of carotenogenic genes in the presence of glucose as a carbon source (9, 10). In yeast, it has been reported that the repressive effect of glucose is transmitted to the cellular machinery by regulatory interactions and signaling pathways at the transcriptional level but is operative even at the posttranscriptional and posttranslational levels (11).

In the canonical mechanism of catabolic repression first described in Saccharomyces cerevisiae (12), the yeast senses intra- and extracellular glucose levels. At high glucose levels, inactive Snf1 kinase leaves the transcription factor Mig1, a DNA-binding protein that is nonphosphorylated and translocates to the nucleus. Here, it recruits a corepressor complex formed by the proteins Cyc8 and Tup1 to carry out the transcriptional repression of the target genes. When glucose becomes limited, Snf1 is activated and phosphorylates Mig1, allowing the cessation of glucose repression and the expression of glucose-repressed genes (11, 12). In agreement with the above, our team showed that X. dendrorhous has a functional catabolic repression mechanism. Moreover, carotenogenesis is inhibited in the presence of glucose, but the inhibition is alleviated in the *mig1*^−/−^, *cyc8*^−/−^, and *tup1*^−/−^ mutant strains (9, 13). Additionally, transcriptomic studies of X. dendrorhous mutant strains *cyc8*^−/−^ and *tup1*^−/−^ have shown that these mutations have a pleiotropic effect, affecting the expression of over 200 genes involved in different biological processes (13).

Previously, Martinez-Moya and collaborators carried out proteomic studies in which they analyzed protein profiles present before and during the induction of carotenogenesis with glucose or succinate carbon sources. In those studies, differentially abundant proteins (DAPs) were identified by two-dimensional polyacrylamide gel electrophoresis (PAGE) followed by mass spectrometry (MS), and the data were correlated with the metabolomic profiles of central metabolic pathways (14, 15). These works showed significant differences in the protein profiles related to the time of analysis and carbon source employed.

To date, the effect of catabolic repression at the protein level in X. dendrorhous has been poorly understood because most of the studies have focused on changes at the transcript level. In the present study, proteomic analyses were complemented with transcriptomic data with the aim of evaluating the metabolic changes associated with the carbon source or with mutations in the *MIG1*, *CYC8*, and *TUP1* genes encoding catabolic regulators Mig1, Cyc8, and Tup1, respectively, during the exponential-growth phase. For this purpose, a quantitative analysis of metabolic pathways was performed based on proteomic iTRAQ (isobaric tags for relative and absolute quantification) data and was correlated with the corresponding transcriptomic sequencing (RNA-seq) data. These analyses demonstrated that this yeast possesses a complex regulatory mechanism involving a convergence between the carbon source and catabolic repression. Additionally, the results suggested cooperative and redundant roles of the repressor Mig1 and the corepressor Cyc8-Tup1 in alleviating the misfunctions in the generated mutant strains, providing valuable information to further reveal the specific forms of regulation occurring at the transcript and protein levels in X. dendrorhous.

## RESULTS

### Influence of different carbon sources on the proteome of X. dendrorhous.

Quantitative proteomics provides the earliest information about a biological system because the measurements focus directly on the biological effects (16). In an attempt to identify the regulatory mechanism associated with the carbon source, we cataloged differences in the abundances of proteins and their transcripts by integrated expression profiling using RNA-seq transcriptomics and high-resolution quantitative iTRAQ proteomics. For this purpose, a wild-type (wt) strain of X. dendrorhous (UCD 67-385) was cultured in the presence of glucose, maltose, or succinate as the sole carbon source, and total RNA or protein samples from the different cultures were harvested.

Transcriptomic analysis was carried out with the transcript sequences of the wild-type strain available in our laboratory (17) as a reference for mapping RNA-seq reads. Values representing numbers of transcripts per million (TPM) were calculated as described previously (18), and differentially expressed genes (DEGs) were identified through the use of four statistical methods with a false-discovery-rate (FDR) *P* value of ≤0.05 and a fold change cutoff criterion. In this analysis, 228 and 465 transcripts were identified as DEGs under the conditions of maltose and succinate treatments, respectively.

**FIG 1:**
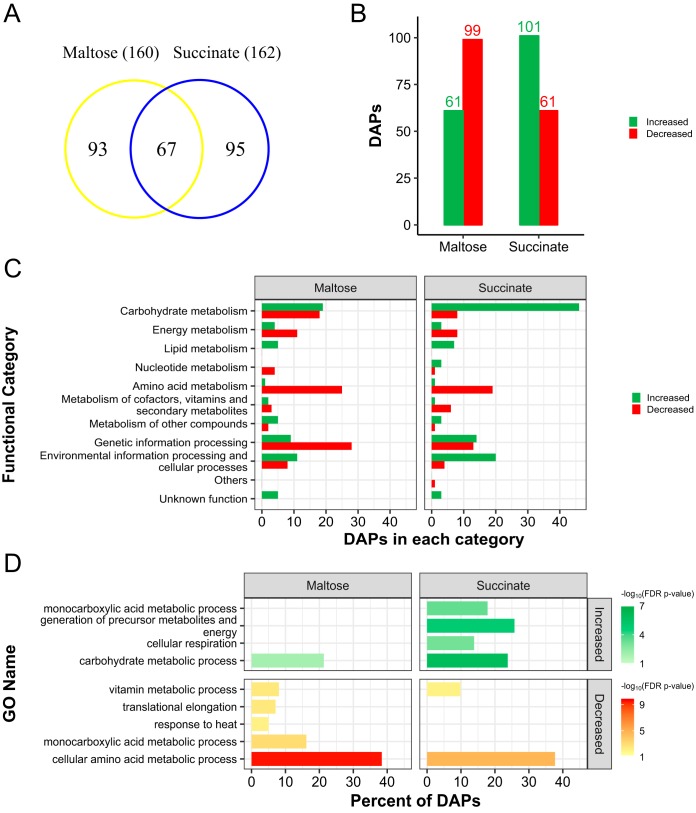
Analysis of differentially abundant proteins (DAPs) under conditions of maltose and succinate treatments of X. dendrorhous. Proteins whose levels changed when maltose or succinate was used as a sole carbon source were compared against those seen when glucose was used as the sole carbon source (FDR *P* value ≤ 0.05). (A) Venn diagram comparing the DAPs identified under both treatment conditions. (B) Data representing DAPs whose levels were increased or decreased by maltose or succinate treatment. (C) Functional classification of DAPs identified based on ortholog annotation in the KEGG public database. (D) Gene Ontology enrichment analysis was carried out with Blast2GO annotation and Fisher’s exact test (FDR *P* value ≤ 0.05), showing highly represented biological processes associated with increased or decreased DAPs under each set of conditions.

For the proteomic analysis, 5,776 peptides were identified that corresponded to 1,161 nonredundant proteins, among which 1,106 were found under conditions of maltose treatment and 1,161 under conditions of succinate treatment. Differentially abundant proteins (DAPs) were defined as proteins that changed their abundance levels with an FDR *P* value of ≤0.05. Comparisons between the samples, taking the glucose treatment as the reference, showed that 14% of the proteins identified under the conditions that employed the alternative carbon sources changed in abundance. Totals of 160 and 162 DAPs were identified under the conditions of maltose and succinate treatments, respectively. Of those, 93 and 95 were found only with maltose and succinate, respectively, and 67 proteins were common under both sets of conditions (Fig. 1A). Compared to the results seen with glucose treatments, 61 and 99 proteins showed increased and decreased levels under conditions of maltose treatment, respectively, while 101 and 61 proteins showed increased and decreased levels with succinate treatment, respectively (Fig. 1B).

To identify which processes were affected by the change in the carbon source, all 255 DAPs were classified according to their biological functions based on Kyoto Encyclopedia of Genes and Genomes (KEGG) annotation (see Table S1C in the supplemental material). KEGG functional classification of the DAPs showed that they were involved in almost every type of biological process (Fig. 1C). DAPs were mainly grouped in the categories of carbohydrate metabolism, genetic information processing, amino acid metabolism, and environmental information processing and cellular processes (37, 37, 26, and 19 DAPs under conditions of maltose treatment and 54, 27, 20, and 24 DAPs under conditions of succinate treatment, respectively), representing over 74% of DAPs under each set of conditions. Gene Ontology enrichment analysis of the biological processes showed that the levels corresponding to the carbohydrate metabolic process and the cellular amino acid metabolic process were increased and decreased, respectively, under both sets of conditions (Fig. 1D). Monocarboxylic acid metabolic processes were found to be increased under conditions of succinate treatment and decreased under conditions of maltose treatment. Other biological processes were uniquely overrepresented under each condition (e.g., cellular respiration was increased with succinate only, while translational elongation was decreased with maltose only).

10.1128/mSphere.00065-20.5 TABLE S1 (A) Yeast strains used in this work. (B) Proteomics design: iTRAQ design and search parameters. (C) Functional classification of DAPs based on KEGG annotation. Download Table S1, DOCX file, 0.02 MB. Copyright © 2020 Martinez-Moya et al. 2020 Martinez-Moya et al. This content is distributed under the terms of the Creative Commons Attribution 4.0 International license.

**FIG 2:**
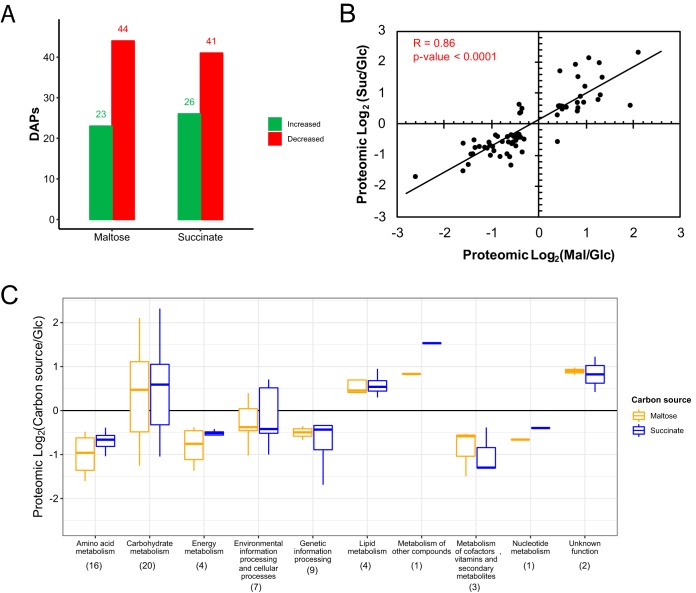
Analysis of common DAPs under conditions of alternative carbon source treatments of X. dendrorhous. Sixty-seven DAPs detected under both maltose (Mal) and succinate (Suc) treatments were analyzed. (A) Common DAPs whose levels were increased or decreased under each set of conditions compared with those seen under the glucose (Glc) treatment conditions. (B) Correlation of proteomic differential abundances compared to the levels seen under glucose treatment conditions for each common DAP. Spearman’s rank correlation coefficient was estimated using GraphPad Prism 5.0.3. The straight diagonal line represents the linear regression of the data. (C) Box plot of the proteomic profile of common DAPs in each functional category under both sets of conditions. Horizontal lines in the boxes represent the medians of each group. The data were analyzed as the base 2 logarithm of fold change with alternative carbon sources over glucose. The numbers in parentheses correspond to the total common DAPs in each category.

On the other hand, among the 67 DAPs common to the two conditions, the expression levels of 23 and 26 were found to be increased by maltose and succinate treatments, respectively, whereas those of 44 and 41 were found to be decreased, respectively (Fig. 2A). There was a positive correlation in the relative abundances of common DAPs seen with each alternative carbon source compared to glucose (Fig. 2B), and the proteomic profile based on their functional classification showed similarity between results associated with the treatments (Fig. 2C). It is noteworthy that the levels of almost all of the common DAPs grouped into amino acid metabolism, genetic information processing, energy metabolism and cofactor metabolism, vitamin, and secondary metabolite functional categories were decreased under conditions of treatments with both carbon sources compared to glucose. Moreover, carbohydrate metabolism and environmental information processing and cellular process functional categories showed a heterogeneous distribution between increased and decreased levels of common DAPs. However, a tendency was observed for the levels of DAPs involved in carbohydrate metabolism to be increased and the levels of those involved in environmental information processing and cellular processes to be decreased.

Moreover, approximately 4% of the DEGs identified in transcriptomic analysis were simultaneously identified as DAPs in proteomic analysis. We focused on the TPM values determined for each DAP, and a positive correlation between transcriptomic and proteomic data was observed for DAPs under each set of conditions (Spearman’s rank correlation coefficients, 0.37 and 0.43 for the maltose and succinate treatments, respectively; *P* value < 0.0001). Thereafter, the data representing the relative abundances of DAPs were complemented with their RNA-seq-based transcript quantification data.

### Mapping of the central metabolic pathways affected by the carbon source.

**FIG 3:**
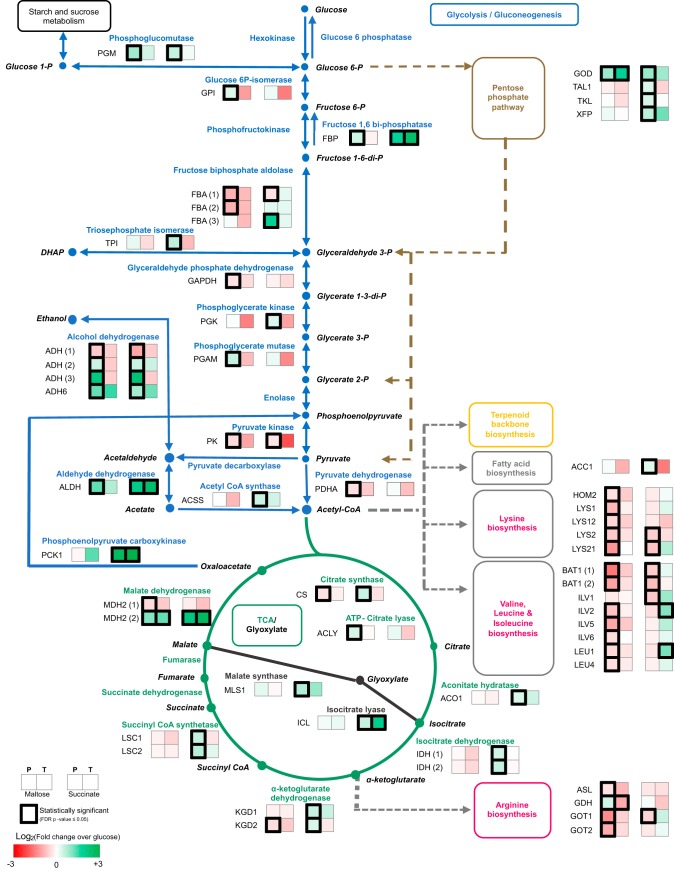
Changes in the protein and transcript levels of enzymes in the central metabolic pathways of X. dendrorhous as an effect of the carbon source. DAPs identified under conditions of maltose or succinate treatments were mapped against central metabolic pathways and compared to those seen under the glucose treatment conditions. Gene names were inferred from KEGG orthologs. P, proteomic; T, transcriptomic.

Carbohydrate metabolism was the most highly represented functional category under both sets of carbon source conditions (see Fig. S1A in the supplemental material). Seventy-one DAPs were found in this category, representing 28% of all DAPs identified under conditions of the use of these alternative carbon sources (Table S2A). These DAPs were mainly mapped to glycolysis/gluconeogenesis, citrate cycle, pyruvate metabolism, glyoxylate and dicarboxylate metabolism, starch and sucrose metabolism, and pentose phosphate (PP) pathways (20, 11, 8, 6, 5, and 4 DAPs, respectively). The effect of the alternative carbon source on the relative abundances of enzymes related to central metabolism was analyzed (Fig. 3).

10.1128/mSphere.00065-20.1 FIG S1 Distribution of DAPs identified under conditions of alternative carbon source treatments and in mutant strains of X. dendrorhous in functional categories. Pie charts show DAPs identified under each set of conditions compared with those produced by the wild-type strain cultured in glucose (i.e., data from the wild-type strain cultured with alternative carbon sources are shown in panel A, and data from the mutant strains cultured with glucose are shown in panel B). Functional classification was performed on the basis of ortholog annotation in the KEGG public database. The sizes of the pie charts are not representative of the total number of DAPs identified under each set of conditions. Download FIG S1, EPS file, 1.4 MB. Copyright © 2020 Martinez-Moya et al. 2020 Martinez-Moya et al. This content is distributed under the terms of the Creative Commons Attribution 4.0 International license.

10.1128/mSphere.00065-20.6 TABLE S2 Proteome and transcriptome comparative and quantitative analyses. (A) Carbon source. (B) Mutant strains. Normalized values are included for every condition; significant data are indicated in bold. Green and red indicate increased and decreased levels, respectively. Download Table S2, XLSX file, 0.1 MB. Copyright © 2020 Martinez-Moya et al. 2020 Martinez-Moya et al. This content is distributed under the terms of the Creative Commons Attribution 4.0 International license.

There was an increase in the number of proteins involved in glycolysis/gluconeogenesis, such as phosphoglucomutase, glucose 6P-isomerase, fructose 1,6-biphosphatase, triosephosphate isomerase, aldehyde dehydrogenase and alcohol dehydrogenases, under both sets of carbon source conditions; a decreased relative abundance was observed for enzymes such as glyceraldehyde phosphate dehydrogenase, pyruvate kinase, and pyruvate dehydrogenase under both conditions. Phosphoenolpyruvate carboxykinase showed an increase in its abundance with succinate and a decrease with maltose. An increase in the relative abundance of proteins involved in the pentose phosphate pathway was observed, with succinate treatment resulting in a more significant difference than maltose treatment. These enzymes were glucose oxidase, transketolase, and xylulose-5-phosphate/fructose-6-phosphate phosphoketolase. There were significant differences in the abundance of transaldolase, which was found to be increased with succinate and decreased with maltose. Regarding the citrate cycle, significant differences between the succinate and maltose treatments were observed, since the abundances of almost all enzymes were found to be increased by succinate and decreased by maltose, including enzymes such as citrate synthase, aconitate hydratase, isocitrate dehydrogenase, and succinyl-coenzyme A (succinyl-CoA) synthetase. In the case of glyoxylate and dicarboxylate metabolism, the abundances of the DAPs, which included isocitrate lyase, glutamine synthetase, and others, mainly increased under both sets of carbon source conditions. In the pyruvate metabolism category, increased levels of DAPs such as d-lactaldehyde dehydrogenase and decreased levels of DAPs such as 2-isopropylmalate dehydrogenase were found under both sets of conditions. Levels of DAPs involved in the assimilation of different carbon sources, such as glycogen synthase and beta-fructofuranosidase (invertase), were found to be mainly increased under both sets of conditions, and those DAPs were mapped to starch and sucrose metabolism and galactose metabolism.

These results showed that similar changes in relative protein abundances were caused by alternative carbon sources, such as maltose and succinate. Since the most highly represented functional category in the DAPs was carbohydrate metabolism and enzymes such as glycogen synthase and beta-fructofuranosidase are known targets of glucose-mediated catabolic repression, these changes may be related to the presence of this regulatory mechanism in X. dendrorhous.

### Effect of mutations in catabolic regulators on the proteome of X. dendrorhous.

In X. dendrorhous, as in other yeasts, the principal factors involved in carbon catabolic repression are the DNA-binding regulator Mig1 and the corepressor complex Cyc8-Tup1 (9, 13). To evaluate the role of these regulators at the proteomic level, the wild-type strain and mutant strains *mig1*^−/−^, *cyc8*^−/−^, and *tup1*^−/−^ (Table S1A) were cultured in glucose, and transcriptomic and proteomic analyses were carried out as before.

**FIG 4:**
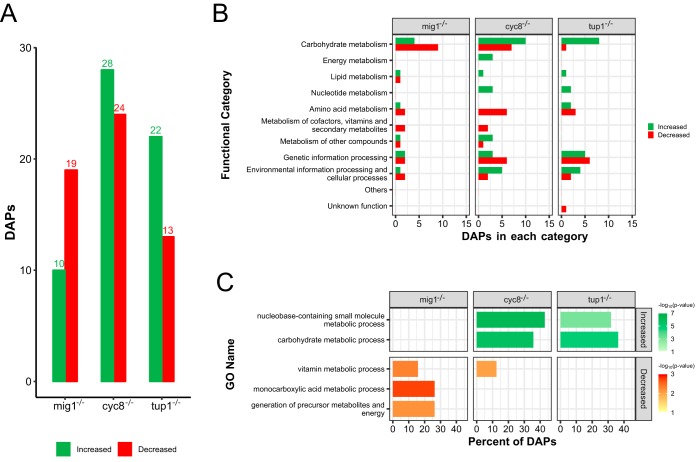
Analysis of DAPs identified in mutant strains of X. dendrorhous. Proteins whose levels changed in the mutant strains *cyc8*^−/−^, *tup1*^−/−^, and *mig1*^−/−^ when glucose was used as the sole carbon source were compared against the wild-type strain under the same conditions (FDR *P* value ≤ 0.05). (A) Data representing DAPs whose levels increased or decreased in each mutant. (B) Functional classification of DAPs identified based on ortholog annotation in the KEGG public database. (C) Gene Ontology enrichment analysis based on Blast2GO annotation and Fisher’s exact test (*P* value ≤ 0.01), showing highly represented biological processes corresponding to DAPs whose levels increased or decreased in each mutant.

In comparison to the wild-type strain, 105, 260, and 19 DEGs were found in the transcriptomic analysis of mutant strains *mig1*^−/−^, *cyc8*^−/−^, and *tup1*^−/−^, respectively. In the proteomic analysis, 1,517, 1,512, and 1,532 proteins were identified in strains *mig1*^−/−^, *cyc8*^−/−^, and *tup1*^−/−^, respectively, and there were 29, 52, and 35 DAPs for each mutant (Fig. 4A; see also Table S2B), which correspond to 89 different proteins. As with the alternative carbon sources, the DAPs found in mutants were classified in almost all functional categories (Fig. S1B). The categories of carbohydrate metabolism, genetic information processing, environmental information processing, and cellular processes and amino acid metabolism were the most highly represented among all DAPs (Fig. 4B), corresponding together to at least 75% of the DAPs in each mutant. Among the Gene Ontology categories, biological, carbohydrate metabolic, and nucleobase-containing-small-molecule metabolic processes were significantly highly represented among the DAPs with increased levels in the *cyc8*^−/−^ and *tup1*^−/−^ mutants, while vitamin metabolic processes were highly represented among the DAPs with decreased levels in the *cyc8*^−/−^ and *mig1*^−/−^ mutants (Fig. 4C). Monocarboxylic acid metabolic processes and generation of precursor metabolites and energy GO terms were uniquely highly represented in the DAPs found with decreased levels in the *mig1*^−/−^ mutant. There was no significant correlation between the transcript quantification of DAPs and their proteomic relative abundances (*P* value < 0.0001).

As with the alternative carbon sources, the most highly represented functional category among the DAPs found in the mutants was carbohydrate metabolism (30 DAPs). The most highly represented pathways in this category were those corresponding to glycolysis/gluconeogenesis, the pentose phosphate pathway, and the citrate cycle. Similar changes in the relative abundances of DAPs involved in those pathways, such as increases in aldehyde dehydrogenase levels and decreases in glyceraldehyde phosphate dehydrogenase levels, were observed in all mutants. In these pathways, the protein abundances shown by the *cyc8*^−/−^ and *tup1*^−/−^ mutants were more similar than those shown by the *mig1*^−/−^ mutant, where almost all DAPs showed decreased levels compared to those of the wild type.

**FIG 5:**
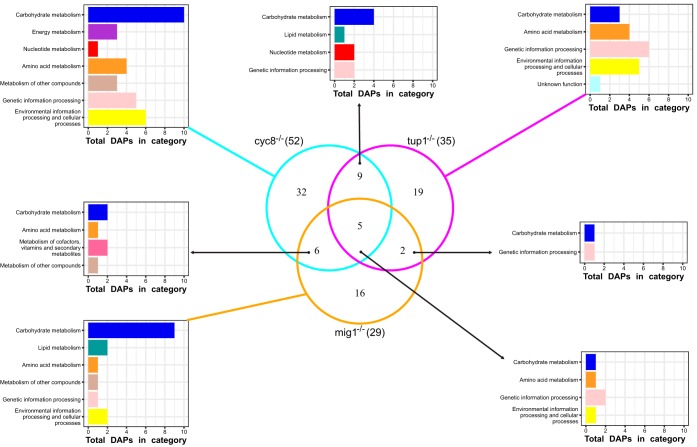
Comparison of DAPs between mutant strains of X. dendrorhous. The Venn diagram shows the number of DAPs shared between at least two of the studied strains and the number of DAPs that were unique for each mutant strain. Bar plots show the DAP classification into functional categories for each comparison.

To evaluate how these regulators may collaborate in glucose-mediated catabolic repression, we compared the DAPs identified among the three mutant strains. It was observed that only 5 DAPs were common to all three mutants (Fig. 5) and that those corresponded to aldehyde dehydrogenase, dihydroxy-acid dehydratase, peptidylpropyl isomerase, cytochrome *c*, and one uncharacterized protein. Mutant strains *cyc8*^−/−^ and *tup1*^−/−^ shared 9 additional DAPs; those 9 DAPs showed mainly increased levels in both strains with respect to the wild type. Moreover, mutant strains *mig1*^−/−^ and *cyc8*^−/−^ shared 6 additional DAPs, which showed similar relative abundances in both mutants, except for pyruvate decarboxylase. The abundance of this enzyme was found to be decreased in strain *mig1*^−/−^ and increased in strain *cyc8*^−/−^. Mutant strains *mig1*^−/−^ and *tup1*^−/−^ shared 2 additional DAPs: heat shock protein 90, which was increased in abundance in both mutant strains, and sorbose reductase, which was decreased in abundance in strain *mig1*^−/−^ and increased in abundance in strain *tup1*^−/−^.

In summary, these results showed that mutations in the *MIG1*, *CYC8*, and *TUP1* regulatory genes mainly affect carbohydrate metabolism at the proteomic level in the presence of glucose compared to the wild-type strain. Additionally, each regulator showed its own set of DAPs, which suggests that these genes may collaborate to control catabolite repression and other regulatory processes in this yeast.

### Convergence between the effects of using alternative carbon sources and mutation of regulators of catabolite repression.

There were some DAPs common to the mutant strains and to the alternative carbon source treatments described previously. Mutant strain *mig1*^−/−^ shared 10 DAPs under both the succinate and the maltose conditions, 2 DAPs with only succinate, and 4 DAPs with only maltose (Fig. S2A). Mutant strain *cyc8*^−/−^ shared 13 DAPs under both conditions, including 8 DAPs with only succinate and 5 DAPs with only maltose (Fig. S2B). Among them, known targets of catabolite repression, such as phosphoenolpyruvate carboxykinase and UDP-glucose dehydrogenase, were found. Moreover, the *tup1*^−/−^ mutant strain shared 6, 4, and 5 DAPs under both succinate and maltose conditions, respectively (Fig. S2C).

10.1128/mSphere.00065-20.2 FIG S2 DAPs shared between the different mutant strains and the wild-type strain of X. dendrorhous cultivated with maltose or succinate as a carbon source. Venn diagrams show the number of DAPs shared between mutant strains *mig1*^−/−^ (a), *cyc8*^−/−^ (b), and *tup1*^−/−^ (c) and the wild-type strain under each of the studied conditions (maltose or succinate). Results of functional classification of DAPs from each comparison are shown in bar charts. Download FIG S2, TIF file, 2.7 MB. Copyright © 2020 Martinez-Moya et al. 2020 Martinez-Moya et al. This content is distributed under the terms of the Creative Commons Attribution 4.0 International license.

**FIG 6:**
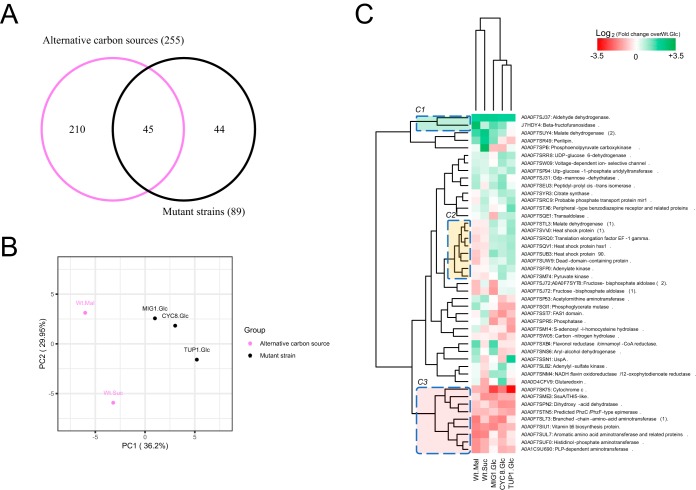
Comparison of DAPs identified under conditions of alternative carbon source treatments and in mutant strains of X. dendrorhous. (A) Venn diagram showing common DAPs identified under conditions of alternative carbon source treatments and in mutant strains cultivated with glucose only. (B) Principal-component analysis of the relative abundances of common DAPs found in both groups. (C) Heat map of the relative abundances of 45 common DAPs identified as shared between the two groups compared against the wild-type strain under conditions of glucose exposure. UniProt identifiers (IDs) and annotation descriptions are shown. Wt.Mal, wild-type strain, maltose treatment; Wt.Suc, wild-type strain, succinate treatment; MIG1. Glc, mutant strain *mig1*^−/−^, glucose treatment; CYC8. Glc, mutant strain *cyc8*^−/−^, glucose treatment; TUP1. Glc, mutant strain *tup1*^−/−^, glucose treatment; Wt. Glc, wild-type strain, glucose treatment.

To obtain a global view of the similarities in the effects observed in the analyses of the alternative carbon sources and mutant strains, the total levels of DAPs identified in the two groups were compared, and it was found that 45 DAPs were common between them (Fig. 6A). Principal-component analysis based on the abundance profile of these DAPs showed grouping of mutant strains separately from alternative carbon sources (Fig. 6B). Interestingly, hierarchical clustering of these DAPs (Fig. 6C) identified clusters that showed similar relative abundances (clusters C1 and C3) and a cluster that showed an inverse profile between the two groups (cluster C2); the abundances were decreased by the alternative carbon sources and increased in the mutant strains. DAPs with similar abundances between groups, including beta-fructofuranosidase (increased) and several proteins related to amino acid metabolism, such as dihydroxy-acid dehydratase and a branched-chain amino acid aminotransferase (both decreased), were found. DAPs with different abundances between groups, including malate dehydrogenase, pyruvate kinase, and adenylate kinase, were also found.

These results show that genes *MIG1*, *CYC8*, and *TUP1* participate in glucose-mediated catabolite repression in X. dendrorhous, since many of the changes observed in the wild-type strain in the presence of the alternative carbon sources were also observed in the strains with mutations of those genes under conditions of glucose exposure. However, a difference was observed between *MIG1* and *CYC8*-*TUP1*, since this complex could participate in the regulation of other processes by either repressing or inducing the expression of genes, as described previously in S. cerevisiae (12).

## DISCUSSION

The natural environment of X. dendrorhous is complex and modulates metabolic responses under conditions of differences in the availability of substrates or in response to oxidative stress, among other factors. Mechanisms mediating adaptations to these conditions, i.e., astaxanthin synthesis, are critical for yeast survival. In the present study, a compressible proteome with complementary transcriptome data was detailed.

First, we identified the metabolic changes associated with the carbon source employed. When glucose is available, the yeast Saccharomyces cerevisiae prefers fermentative metabolism even in the presence of oxygen and represses respiration, the use of alternative carbon sources, and gluconeogenesis (11). In accordance with our previous results (15) for X. dendrorhous, we observed an expected pattern associated with the assimilation pathway; i.e., in succinate, there are upregulations of the glyoxylate cycle, PP pathway, and tricarboxylic acid (TCA) cycle (Fig. 3). Indeed, target proteins for transcriptional catabolic repression, such as fructose 1-6-biphosphatase and aldehyde dehydrogenase (11), were regulated under both sets of carbon source conditions, and malate synthase, isocitrate lyase, and acetyl-CoA synthase were upregulated only with succinate as a carbon source. Notably, for amino acid biosynthesis, we observed downregulation of proteins under conditions of maltose and succinate exposure (DEGs and DAPs identified) (Fig. 3). This may be related to the different levels of regulation of this pathway, for instance, at the posttranslational level (19). For S. cerevisiae, the posttranscriptional and posttranslational effects of catabolic repression balance cellular energy by inactivating expensive cellular processes, such as amino acid and lipid biosynthesis (11).

Notably, in contrast to our previous results (15), we did not identify the regulation of carotenogenic proteins under our experimental conditions. This can be explained by the fact that it is very difficult to recover carotenogenic proteins at the time of harvest. In the case of transcripts, it was reported previously that an increment was observable at the end of the exponential-growth phase of X. dendrorhous (5, 6).

To correlate the effect of assimilation of different carbon sources on central metabolism, we analyzed the proteome and transcriptome of strains *cyc8*^−/−^, *tup1*^−/−^, and *mig1*^−/−^, encoding principal transcriptional regulators. Glucose repression and derepression essentially concern genes involved in oxidative metabolism and the TCA cycle, genes coding for the metabolism of alternative carbon sources, or genes coding for gluconeogenesis (11–20). The most common mechanisms regulating gene products involve a decrease in transcription or translation at the gene level or an increase in protein degradation at the protein level (12).

Comparing the DAPs identified in alternative carbon sources and mutant strains, we observed a moderate effect on central metabolism (Fig. 6A). For 45 DAPs that were common between the two groups, we observed that all shared proteins in cluster C1 corresponded to targets associated with transcriptional repression (aldehyde dehydrogenase, invertase, and malate dehydrogenase) and that proteins of cluster C3 corresponded to expensive energetic processes in the cell, such as vitamin and amino acid biosynthesis. For cluster C2, differentiation between the carbon source and mutant treatments was clear. However, in general, there were close relationships among the protein profiles of Cyc8, Tup1, and Mig1 (Fig. 6C).

To date, Mig1 has been described as a principal transcriptional regulator for catabolite repression in X. dendrorhous (9) but has also been reported to be associated with the response to osmotic stress in S. cerevisiae (21). We observed that invertase, malate dehydrogenase, and aldehyde dehydrogenase were derepressed in the absence of Mig1 but that fructose biphosphate aldolase and phosphoenolpyruvate carboxykinase were repressed. Contrasting results have been found in previous studies in which the inactivation or deletion of Mig1 partially eliminated glucose repression, and it is probable that a second repressor persists as an independent glucose control mechanism (22). Other evidence of a general role for Mig1 has been described in Kluyveromyces marxianus wherein histidine biosynthesis was positively regulated (23), in Pichia pastoris for autophagy and peroxisome biogenesis (24), and in S. cerevisiae for acetate metabolism (25).

Notably, the similar profiles of DAPs shared between mutants *cyc8*^−/−^ and *tup1*^−/−^, for instance, showing upregulated glycolysis and gluconeogenesis proteins and downregulated amino acid biosynthesis proteins, suggest opposite and collaborative roles according to the environmental conditions. Indeed, it has been reported previously that the Cyc8-Tup1 complex showed antagonistic and activator effects (26–28), but it is necessary to elucidate this behavior and its effect on carotenogenesis.

Concerning the function of Cyc8 and Tup1, a corepressor inhibits transcription that is mediated by factors distinct from the recruiting protein; i.e., inhibiting activation is mechanistically distinct from repression (29). Cyc8-Tup1 represses transcription via multiple mechanisms (30), modifying the chromatin structure via recruitment of histone deacetylases (HDACs), directing the positioning of nucleosomes, inhibiting the general transcription machinery via direct interactions, and masking the activation domains of DNA-binding proteins to prevent the recruitment of coactivators. On the basis of our results, we observed more plasticity due to Cyc8 and Tup1 deletions than due to deletion of the repressor Mig1. The Cyc8 and Tup1 corepressors represent a general and versatile stress regulatory complex that permits rapid transitions between repressed and activated states by several mechanisms (30). The Cyc8-Tup1 coactivator plays a role at the transcriptional level for amino acid transporter genes (*AAT*) via Stp1/2p activators in S. cerevisiae (27). Studies have suggested that, in many cases, Tup1 is the protein that carries out repression and Cyc8 mediates the interaction with DNA-binding repressor proteins (29).

Three different domains of Tup1 have been identified as participating in the control of the expression of different target genes, localized in the N-terminal, middle, and C-terminal regions of the protein (31). Specifically, overexpression of 200 amino acids at the N terminus of Tup1 repressed the *SUC2*, *RNR2*, and *OLE1* genes and, consequently, deletion of the C-terminal repression domain alleviated repression of all tested target genes (31). Interestingly, although Tup1 is a transcriptional repressor, a previous study showed that complete deletion of *TUP1* was not beneficial for glucose derepression to facilitate maltose metabolism in S. cerevisiae (32). Thus, it has been documented that *TUP1* overexpression and deletion have opposing effects on maltose metabolism, which is consistent with our results. Indeed, in S. cerevisiae, deletion of Mig1 and/or Cyc8 to facilitate maltose metabolism has been reported to be effective (32).

The Mig1 repressor and Cyc8-Tup1 corepressor complex control gluconeogenic genes such as *FBP1* (encoding fructose-1,6-biphosphatase 1) and *ICL1* (encoding isocitrate lyase 1). It has been demonstrated that this corepressor complex can be converted to the coactivator Cti-Cyc8-Tup1, playing a principal role in the transcriptional activation of *FBP1* and *ICL1* (33, 34). In other work, it was observed previously that in S. cerevisiae, dysfunction of Cyc8-Tup1 confers the ability to assimilate mannitol to produce ethanol (35). All previously cited works showed evidence of collaborative, opposite, or redundant roles for the Mig1 repressor and Cyc8-Tup1 corepressor complex.

According to our results, one possible explanation is that an unknown novel repressor/activator, or another uncharacterized protein, functions together with Mig1 and Cyc8-Tup1 to control carbon source effects in X. dendrorhous. Alternatively, repressive mechanisms can work cooperatively to regulate genes involved in carbon source assimilation and metabolism; e.g., Cyc8-Tup1 represses transcription (30). As stated above, the various corepressors have distinct and overlapping functions. Recently, a phenomenon called epigenetic transcriptional memory showed that expressed inducible genes can remain poised for faster reactivation for multiple cell divisions; i.e., in S. cerevisiae, the GAL genes showed faster reactivation for up to seven generations after being repressed (36) or showed different growth behaviors when their carbon sources were alternated (37).

In summary, in this study, we examined the convergence between the metabolic influence of the carbon source in X. dendrorhous and the catabolic repression effect in mutant strains Cyc8, Tup1, and Mig1. We observed a variation of 14% in protein abundance due to the carbon source treatments compared with the mutant treatments, for which only 2% variation was observed. Transcriptome data did not show a direct correlation with protein abundance, indicating several points of regulation, such as RNA degradation, enzymatic activity, and posttranslational and epigenetic factors.

## MATERIALS AND METHODS

### Strains and culture conditions.

X. dendrorhous strains (see Table S1A in the supplemental material) were grown at 22°C with constant agitation in YM medium (0.3% yeast extract, 0.3% malt extract, and 0.5% peptone) supplemented, when indicated, with 1% glucose (YM-1% glucose), or in Vogel minimal medium supplemented with 2% glucose, 2% succinate, or 2% maltose. The cells were grown to the early exponential-growth phase (optical density at 600 nm [OD_600_] of 1 to 2.5) and then collected for both transcriptomic and proteomic experiments. Samples were harvested through centrifugation, and the pellet was washed twice with ice-cold water, centrifuged at 5,000 × *g* for 10 min at 4°C, and stored at −80°C until further analysis.

X. dendrorhous mutant strains with a mutation in gene *MIG1* (KX384897), gene *CYC8* (KX517902), or gene *TUP1* (KX517903) were derived from wild-type strain UCD 67-385 (ATCC 24230) and were obtained via homologous recombination performed to replace the corresponding gene with a cassette that conferred resistance to an antibiotic (38). For the *MIG1* gene mutation, plasmid pDel_gMIG1 (see Fig. S3 in the supplemental material) was constructed by inserting at the EcoRV site of the pBluescript II SK(-) a 1,356-bp fragment that was previously obtained by overlap extension PCR analysis of the adjacent sequences upstream (621 bp) and downstream (729 bp) of the open reading frame (ORF) of the *MIG1* gene and replacing this ORF with an HpaI restriction site. The upstream and downstream fragments were subjected to PCR amplification using DNA of the wild-type strain as a template and primer pairs del_gMIG1-Fw (5′-GTAGGTCCGGGTGTGTGTATA-3′) plus del_gMIG1HpaI-Rv (5′-AGACATCCTTCGTTAACTATCGAATGTTGTTCCTGCC-3′) and del_gMIG1HpaI-Fw (5′-AACATTCGATAGTTAACGAAGGATGTCTGGTTACTTC-3′) plus del_gMIG1-Rv (5′-GAACAGATGCGCTGGTCTACA-3′), respectively.

10.1128/mSphere.00065-20.3 FIG S3 Plasmid used for the construction of the *mig1*^−/−^ gene deletion mutant of X. dendrorhous. (Top) pDel_gMIG1 pBluescript II SK(-) containing a 1,236-bp fragment obtained by overlap extension PCR of the adjacent sequences upstream (609 bp) and downstream (729 bp) of the ORF of the *MIG1* gene, which was replaced by an HpaI site used for the construction of the plasmids pDel_MIG1-Hyg (middle) and pDel_MIG1-Zeo (bottom). The yellow bar corresponds to pBluescript II SK(-) sequences. PEF, EF-1a promoter; gpdT, *GPD* transcription terminator of X. dendrorhous. Download FIG S3, EPS file, 1.8 MB. Copyright © 2020 Martinez-Moya et al. 2020 Martinez-Moya et al. This content is distributed under the terms of the Creative Commons Attribution 4.0 International license.

Plasmids pDel_MIG1-Zeo and pDel_MIG1-Hyg were constructed by the insertion, into the HpaI restriction site of pDel_gMIG1, of modules that confer resistance to zeocin and to hygromycin B, respectively. The transformation of X. dendrorhous occurred with the Del_gMIG1-Zeo and Del_gMIG1-Hyg modules, respectively. The transformant DNA containing each module was released from the respective pDel_gMIG-Zeo and pDel_gMIG-Hyg plasmids by digestion with the SpeI and ClaI restriction enzymes. The homozygote *mig1*^−/−^ deletion mutant strain had resistance to both zeocin and hygromycin B (Fig. S3).

### RNA extraction and transcriptome sequencing.

The total RNA extraction protocol was performed according to a modified protocol previously described (13).

Transcriptomes of the wild-type and mutant strains were obtained from RNA samples taken from cultures during the early exponential-growth phase (36 h of culture) using minimal medium supplemented with 2% glucose, 2% succinate, or 2% maltose. When the samples were taken, 1% glucose was present in the culture media. RNA-seq was performed by Macrogen Inc. (Seoul, South Korea) using an Illumina HiSeq2000 system with a 100-bp paired-end library as described previously (17). The raw transcriptomic data were analyzed with CLC Genomics Workbench 11.0, and the gene expression level of those uniquely mapped reads was estimated by calculating their TPM values (18). DEGs were identified by four statistical methods, including those using the DEseq2 (39), edgeR (40), and EBSeq packages available in Bioconductor Repository (www.bioconductor.org) and the CLC Genomics Workbench 11.0 method, which itself was used with a *P* value corrected for the FDR (41). In this study, those genes with absolute log_2_ TPM ratio values greater than 1 and FDR values of ≤0.05 were significantly expressed. To facilitate correlation analysis with protein data, all significant values were normalized using a log_2_ method (i.e., for analysis of TPM-other carbon source/TPM-glucose or TPM-mutant/TPM-wt correlations).

### Protein extraction.

Protein extracts were obtained from three biological replicates (using different independent cultures) (Fig. S4). For extraction, each cell pellet was treated with 1 volume of lysis buffer [100 mM sodium bicarbonate, 0.5% Triton X-100, 1 mM phenylmethylsulfonyl fluoride (PMSF), 2% protease inhibition cocktail (Promega), 2 mM Tris(2-carboxyethyl)phosphine hydrochloride (TCEP)] and glass beads (0.5 mm in diameter). Seven cycles of disruption of 30 s each were performed using a Mini-Beadbeater-16 “cell grinder” (BioSpec). Between disruption cycles, the samples were incubated on ice for 1 min. Then, centrifugation was performed at 4°C for 20 min at 14,000 rpm, and the supernatant was recovered. The supernatants were treated for 30 min at room temperature with a solution containing DNase and RNase. The proteins were analyzed in acrylamide gels under denaturant conditions (SDS-PAGE) and quantified using a Pierce bicinchoninic acid (BCA) protein assay kit (Thermo Scientific). Protein samples were lyophilized and stored until analysis.

10.1128/mSphere.00065-20.4 FIG S4 Growth profile of wild-type and mutant strains of X. dendrorhous under experimental conditions. All strains were grown in minimal medium supplemented with glucose (2%; continuous lines). Also, the wild-type strain was grown in minimal medium supplemented with maltose or succinate (both 2%; dashed lines). Samples were collected at the early exponential-growth phase (OD_600_ of 1 to 2.5) for proteomic analyses. Wt, wild-type strain; *cyc8* ^−/−^, mutant strain *cyc*8 ^−/−^; *tup1*^−/−^, mutant strain *tup1*^−/−^; *mig1*^−/−^, mutant strain *mig1*^−/−^; Glc, glucose; Mal, maltose; Suc, succinate. Download FIG S4, EPS file, 1.4 MB. Copyright © 2020 Martinez-Moya et al. 2020 Martinez-Moya et al. This content is distributed under the terms of the Creative Commons Attribution 4.0 International license.

### Proteomic analyses.

Proteomics analyses were performed at the Proteomics Core Facility of the National Center of Biotechnology (CNB-CSIC; Madrid, Spain). The different protein samples were analyzed by iTRAQ technology coupled to mass spectrometry (MS). Two iTRAQ 8-plex experiments were conducted, each containing all sample types, for two replicates (Table S1B). For comparison purposes, the proteins obtained from the wild-type strain with glucose as the carbon source were used as a reference.

Tandem MS (MS/MS) spectra were searched against MASCOT (Matrix Science, v.2.5), OMSSA (NCBI, v.2.1.9), X!Tandem2 (TheGPM; v.win-13-02-01-1), X!Tandem2 with k-score plugin (LabKey Software, v.2.3-7806), Myrimatch (Vanderbilt University, v.2.1), or MS-GF+ (CCMS-NIGMS, v. Beta v10072) databases and were arranged by FDR significance (FDR ≤ 0.01) according to the peptides and proteins identified. The search parameters are detailed in the supplemental material (Table S1B).

For quantitative analysis, only those peptides identified with an FDR value of ≤0.01 were used. For each inferred protein, the peptides were classified according to their abundance in quantitative groups and then were normalized by log_2_ transformation. DAPs were defined as proteins that changed their abundance levels with an FDR *P* value of ≤0.05 with respect to the control group. The protein quantitation information is provided in Table S2.

### Bioinformatic and statistical analysis.

Functional annotation of DAPs was carried out through the Kyoto Encyclopedia of Genes and Genomes via BlastKOALA (42) and kofamKOALA (43). KEGG mapping was carried out using the KO Database of Molecular Functions (reference hierarchy), and functional classification was performed as described in Table S1C. Gene Ontology annotation of all proteins quantified in each iTRAQ experiment was carried out with the Blast2GO suite (44), filtering GO annotation data to basidiomycete taxa and reducing these results through yeast GO-Slim. Enrichment analysis of biological processes was carried out by applying Fisher’s exact test comparing DAP annotations against all proteins identified.

Principal-component analysis, heat map construction, and hierarchical clustering were all carried out with R using default packages. The Lilliefors test for normality (*P* value < 0.01) was also carried out in R using the DescTools package. Correlations between each pair of transcriptomic and proteomic data sets were assessed using Spearman’s rank correlation coefficient with GraphPad Prism 5.0.3 (*P* value < 0.0001).

### Data availability.

All relevant data are included in the paper and its supplemental files.
