# Supplementary material for: Convergence between Regulation of Carbon Utilization and Catabolic Repression in Xanthophyllomyces dendrorhous
Source: mSphere. 2020 Apr 1;5(2):e00065-20. doi: 10.1128/mSphere.00065-20 (PMC7113583; doi:10.1128/mSphere.00065-20)
Supplement: TABLE S1 [file mSphere.00065-20-st001.docx]

**Table S1. Material and Methods**

**A. Yeast strains used in this work.**

| Strain | Description | Reference or source |
| --- | --- | --- |
| UCD 67–385 | Diploid wild-type strain (Hyg^S^ and Zeo^S^) | ATCC 24230; Miller et al., 1976 |
| Δ*mig1^-/-^* | Hyg^R^ and Zeo^R^ homozygous mutant (*mig1^−^*/*mig1^−^)* that derives from UCD 67–385. A *MIG*1 allele was replaced by a module that confers resistance to hygromycin B and the other allele by a module that confers resistance to zeocin (knock-out mutation). | This work |
| *cyc8^-/-^* | Hyg^R^ and Zeo^R^ homozygous mutant (*cyc8*^−^/*cyc8*^−^) that derives from UCD 67–385. A *CYC8* allele was interrupted by a module that confers resistance to hygromycin B and the other allele by a module that confers resistance to zeocin | Córdova et al., 2016 |
| *tup1^-/-^* | Hyg^R^ and Zeo^R^ homozygous mutant (*tup1*^−^/*tup1*^−^) that derives from UCD 67–385. A *TUP1* allele was interrupted by a module that confers resistance to hygromycin B and the other allele by a module that confers resistance to zeocin | Alcaíno et al., 2016 |

Hyg^S^ sensitive to hygromycin B, Hyg^R^ resistant to hygromycin B, Zeo^S^ sensitive to zeocin, Zeo^R^ resistant to zeocin, ATCC American Type Culture Collection

**B. Proteomics design: iTRAQ design and search parameters.**

| **iTRAQ design** | | | |  |  |  |  |
| --- | --- | --- | --- | --- | --- | --- | --- |
| iTRAQ 1 | |  | | iTRAQ 2 |  |  |  |
| Ion reporter | | Strain-media-carbon source | | Ion reporter | Strain-media-carbon source |  |  |
| 113 | | *wt* - MMv-glc | | 113 | *wt* - MMv-glc |  |  |
| 114 | | *wt* - MMv-mal | | 114 | *wt* - MMv-glc |  |  |
| 115 | | *wt* - MMv-suc | | 115 | *tup1^-/-^* - MMv-glc |  |  |
| 116 | | *wt* - YM | | 116 | *tup1^-/-^* - MMv-glc |  |  |
| 117 | | *wt* - MMv-glc | | 117 | *cyc8^-/-^* - MMv-glc |  |  |
| 118 | | *wt* - MMv-mal | | 118 | *cyc8^-/-^* - MMv-glc |  |  |
| 119 | | *wt* - MMv-suc | | 119 | Δ*mig1^-/-^* - MMv-glc |  |  |
| 121 | | *wt* - YM | | 121 | Δ*mig1^-/-^* - MMv-glc |  |  |
| Wt, wild type | |  | |  |  |  |  |
| **Search parameters** | | | |  |  | | |
| mode | site | | type | | description | |  |
| fixed | C | | chemical | | L-cysteine methyl disulfide | |  |
| variable | M | | chemical | | methionine oxidation | |  |
| variable | Q at peptide N-terminus | | chemical | | pyroglutamic acid from glutamine | |  |
| variable | E at peptide N-terminus | | chemical | | pyroglutamic acid from glutamic acid | |  |
| variable | protein N-terminus | | PTM | | acetylation of protein N-terminus | |  |
| variable | peptide N-terminus | | isobaric tagging reagent | | iTRAQ-8plex | |  |
| variable | peptide N-terminus | | isobaric tagging reagent | | iTRAQ-8plex | |  |
| variable | K | | isobaric tagging reagent | | iTRAQ-8plex | |  |
| variable | K | | isobaric tagging reagent | | iTRAQ-8plex | |  |
| variable | Y | | isobaric tagging reagent | | iTRAQ-8plex | |  |
| variable | Y | | isobaric tagging reagent | | iTRAQ-8plex | |  |

**C. Functional classification of DAPs based on KEGG annotation.**

| **Functional Category** | **KEGG Pathway** |
| --- | --- |
| Carbohydrate metabolism | - 09101 Carbohydrate metabolism. - 09107 Glycan biosynthesis and metabolism. - 09191 Unclassified: metabolism (Partial: Carbohydrate metabolism & Glycan metabolism). |
| Energy metabolism | - 09102 Energy metabolism. - 09191 Unclassified: metabolism (Partial: Energy metabolism). |
| Lipid metabolism | - 09103 Lipid metabolism. - 09191 Unclassified: metabolism (Partial: Lipid metabolism). |
| Nucleotide metabolism | - 09104 Nucleotide metabolism. - 09191 Unclassified: metabolism (Partial: Nucleotide metabolism). |
| Amino acid metabolism | - 09105 Amino acid metabolism. - 09106 Metabolism of other amino acids. - 09191 Unclassified: metabolism (Partial: Amino acid metabolism). |
| Metabolism of cofactors, vitamins and secondary metabolites | - 09108 Metabolism of cofactors and vitamins. - 09109 Metabolism of terpenoids and polyketides. - 09110 Biosynthesis of other secondary metabolites. - 09191 Unclassified: metabolism (Partial: Cofactors metabolism & Secondary metabolism). |
| Metabolism of other compounds | - 09111 Xenobiotics biodegradation and metabolism. - 09181 Protein families: metabolism. - 09191 Unclassified: metabolism (Partial: Enzymes with EC number & others). |
| Genetic information processing | - 09120 Genetic information processing. - 09182 Protein families: genetic information processing. - 09192 Unclassified: genetic information processing. |
| Environmental information processing and cellular processes | - 09130 Environmental information processing. - 09140 Cellular processes. - 09183 Protein families: signaling and cellular processes. - 09193 Unclassified: signaling and cellular processes. |
| Others | - 09150 Organismal systems. - 09160 Human diseases. |
| Unknown function | - Poorly characterized. - Without K-number. |
