# Supplementary figures and images for: Convergence between Regulation of Carbon Utilization and Catabolic Repression in Xanthophyllomyces dendrorhous
Source: mSphere. 2020 Apr 1;5(2):e00065-20. doi: 10.1128/mSphere.00065-20 (PMC7113583; doi:10.1128/mSphere.00065-20)

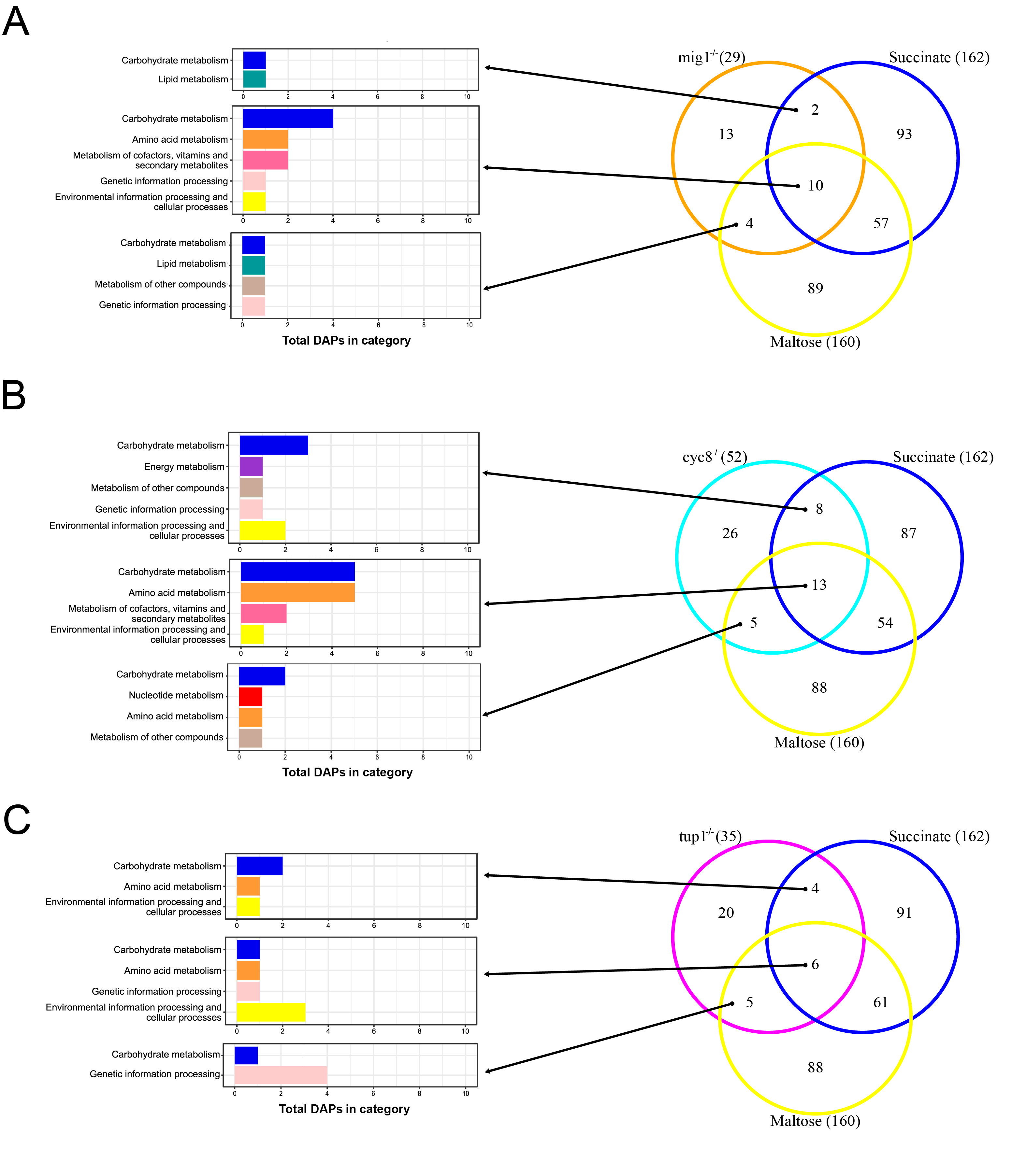

Supplement: FIG S2 [file mSphere.00065-20-sf002.tif]
